# Strategies to Overcome Failures in T-Cell Immunotherapies by Targeting PI3K-δ and –γ

**DOI:** 10.3389/fimmu.2021.718621

**Published:** 2021-08-26

**Authors:** Sanjay Chandrasekaran, Christopher Ronald Funk, Troy Kleber, Chrystal M. Paulos, Mala Shanmugam, Edmund K. Waller

**Affiliations:** ^1^Department of Hematology and Medical Oncology, Winship Cancer Institute at Emory University, Atlanta, GA, United States; ^2^Department of Surgery/Microbiology & Immunology, Winship Cancer Institute at Emory University, Atlanta, GA, United States

**Keywords:** adoptive cell immunotherapy, TIL (tumor infiltrating lymphocytes), CAR T cancer therapy, immune checkpoint inhibition (ICI), PI3K delta, PI3K gamma, T cell differentiation

## Abstract

PI3K-δ and PI3K-γ are critical regulators of T-cell differentiation, senescence, and metabolism. PI3K-δ and PI3K-γ signaling can contribute to T-cell inhibition *via* intrinsic mechanisms and regulation of suppressor cell populations, including regulatory T-cells and myeloid derived suppressor cells in the tumor. We examine an exciting new role for using selective inhibitors of the PI3K δ- and γ-isoforms as modulators of T-cell phenotype and function in immunotherapy. Herein we review the current literature on the implications of PI3K-δ and -γ inhibition in T-cell biology, discuss existing challenges in adoptive T-cell therapies and checkpoint blockade inhibitors, and highlight ongoing efforts and future directions to incorporate PI3K-δ and PI3K-γ as synergistic T-cell modulators in immunotherapy.

## Introduction

T-cell based immunotherapies aim to reinvigorate immunity against malignant cells either *via* infusion of effector T-cells or activation of existing T-cells in the body. Here, we provide an overview of the mechanism of PI3K signaling in T-cells, particularly PI3K-δ and -γ, down-stream of T-cell receptor activation. Particular attention is given to how inhibition of PI3K-δ and -γ signaling with drug inhibitors regulates and activates pathways related to T-cell proliferation, differentiation, senescence, exhaustion, and metabolism. In this context, we consider the current state of therapies targeting T-cell immune checkpoint pathways and the effects of synergizing PI3K inhibition with immune checkpoint inhibitors (ICIs) to re-model the activity of T-cells and other immunosuppressive cells in the tumor microenvironment (TME). We also consider PI3K-δ and -γ signaling and inhibition in the context of Adoptive T-cell Transfer (ACT) therapies. ACT therapies entail harvesting a patient’s immune cells, culturing and potentially modifying them *ex vivo*, and reinfusing them back into the patient. Within ACT, three commonly utilized approaches used for patients are chimeric antigen receptor T-cell (CART), TCR (T-cell Receptor) therapy, and tumor infiltrating lymphocyte (TIL) therapies. Manufacturing ACT products are costly, time consuming, technically challenging, and clinical responses are promising but not consistently achieved (1, 2). We subsequently review the current preclinical and clinical progress with ACT therapies, highlight notable differences between their manufacturing processes, and discuss specific areas for process improvement in the context of surrogate measures of clinical outcomes. Finally, while reviewing current limitations in ACT, we discuss how PI3K-δ and -γ are promising pharmacological targets for improving T-cell response in ACT given their ubiquitous expression in T-cells.

## Overview Of Pi3k Signaling In T-Cells

PI3K proteins are divided into class IA, IB, II, and III and are named by order of discovery. Class IA subunits include -α, -β and -δ, and are generally phosphorylated by receptor tyrosine kinases (RTKs). Class IB is comprised of the PI3K-γ isoform and is expressed and co-localized with G-protein coupled receptors (GPCRs). In healthy tissue, the PI3K-α and -β isoforms are ubiquitously expressed whereas the PI3K-δ and -γ isoforms are mainly expressed in hematopoietic cells (3, 4). PI3K-δ and/or -γ inhibition and knockout in lymphocytes reduces cytokine-mediated chemotaxis. In particular, knockout of PI3K-γ reduced T-cell migration, while PI3K-δ knockout generated deficiencies in B cell chemotaxis (5).

When over-expressed in cancers, PI3K-α and -β drive tumor growth and metastasis while expression of PI3K-δ and -γ in hematopoietic cells regulates immune cell activity, especially lymphocyte and myeloid cell differentiation and activation (6–8). In many solid tumor malignancies, including breast cancer, lung, head and neck cancer, and melanoma, increased activity within the PI3K pathway occurs through activating mutations and gene amplifications in *PIK3CA* (PI3K-α), or loss of expression of the PI3K tumor suppressor, PTEN (8–15). Efforts to develop pan-PI3K inhibitors into successful anti-cancer therapy have been stymied by low response rates to PI3K inhibitors and significant toxicities (16–23). Unlike the use of PI3K inhibitors in solid tumor malignancies, the anti-tumor effects of PI3K inhibition in lymphoid malignancies are not dependent on gene mutations, amplifications, or deletions within the PI3K pathway. Instead, PI3K-δ and -γ inhibitors exert direct inhibitory effects on lymphoid cancer cell survival, and indirect effects by targeting survival and homing of normal lymphoid and myeloid cells into the TME (24–26). These bi-functional properties are a result of PI3K-δ and -γ signaling regulating activation and differentiation of normal B- and T-cells while simultaneously limiting the proliferation of the cancer counterparts of normal lymphoid cells following their neoplastic transformation.

FDA approved therapies now exist for relapsed B-cell malignancies like CLL and Follicular Lymphoma (FL) with idelalisib (PI3K-δ), duvelisib (PI3K-δ/γ), and copanlisib (PI3K-α/δ) (16, 27–31). Clinical trials are ongoing in Richter Syndrome or transformed FL, Relapsed/Refractory T-cell Lymphomas, Relapsed or Refractory Peripheral T-cell Lymphoma (PTCL), ALL, maintenance post autologous transplant for T-cell and indolent B cell lymphomas, DLBCL, Mantle Cell lymphoma, and Marginal Zone Lymphoma (NCT03892044, NCT02783625, NCT03372057, NCT04331119, NCT03742323, NCT03133221, NCT04233697, NCT04263584, NCT03877055, and NCT03474744) (32).

The direct anti-cancer activity of PI3K-δ and -γ inhibitors in hematologic malignancies occurs by inhibiting the proliferation of malignant cells with a simultaneous indirect effect on normal hematopoietic cells that is related to limiting the immune suppressive properties of the TME (8, 33, 34). Studies in healthy T-cell biology have unequivocally demonstrated that PI3K signaling drives T-cell differentiation and senescence and supports immune homeostasis by Tregs (3, 6, 35, 36).

### TCR Complexes Signals Through PI3K-δ and -γ

**Figure 1:**
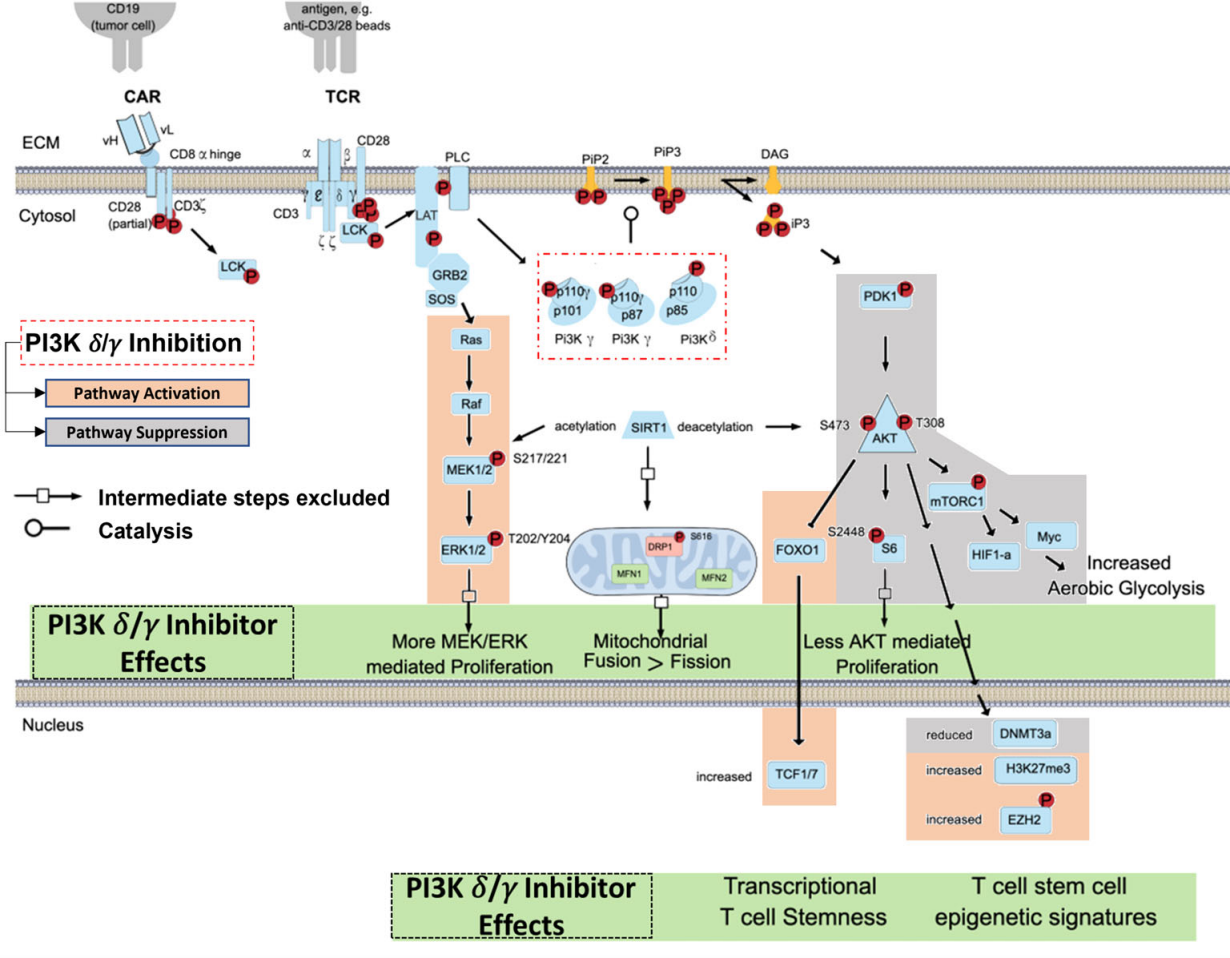
Blocking TCR/CAR Mediated Activation of PI3K Signaling in T cells. TCR/CAR binding by antigen results in downstream signaling through PI3K *δ/γ*, AKT, and mTORC1. This signal cascade promotes AKT mediated proliferation, aerobic glycolysis, and FOXO1 inhibition, and loss of TCF1/7 and the stem-cell like epigenetic markers phosphorylated EZH2 and H3K27me3, leading effector T-cell generation. PI3K *δ/γ* reverse these effects and in turn increasing proliferative signaling through MEK and ERK, increase mitochondrial fusion, and promote epigenetic changes associated with T cell stemness.

The TCR/CD3 complex and related CAR constructs work in concert with co-stimulatory signals like CD28 to induce T-cell activation *via* PI3K pathway signaling (37, 38). In brief, CD3-mediated signaling through Lck, LAT, and PLC protein leads to phosphorylation of the p110δ and p110γ subunits of the PI3K-δ and PI3K-γ isoforms (**Figure 1**). The p110δ and p110γ subunits work by phosphorylating phosphatidylinositol 4,5 bisphosphate (PIP2) to yield phosphatidylinositol 3, 4,5 triphosphate (PIP3), which permits anchorage and association of cytosolic proteins near the lipid bilayer to facilitate downstream signal transduction (39). PIP3 thus functions as a second messenger initiating multiple signaling cascades, most notably facilitating the phosphorylation of AKT by PDK1 which subsequently leads to downstream survival and differentiation signals secondary to activation of the mechanistic target of rapamycin 1 (mTORC1) (40, 41) (**Figure 1**). We have shown that inhibition of PI3K-δ and -γ in T-cells with duvelisib simultaneously activates signaling through the RAS/RAF/MEK pathway (42).

**Figure 2:**
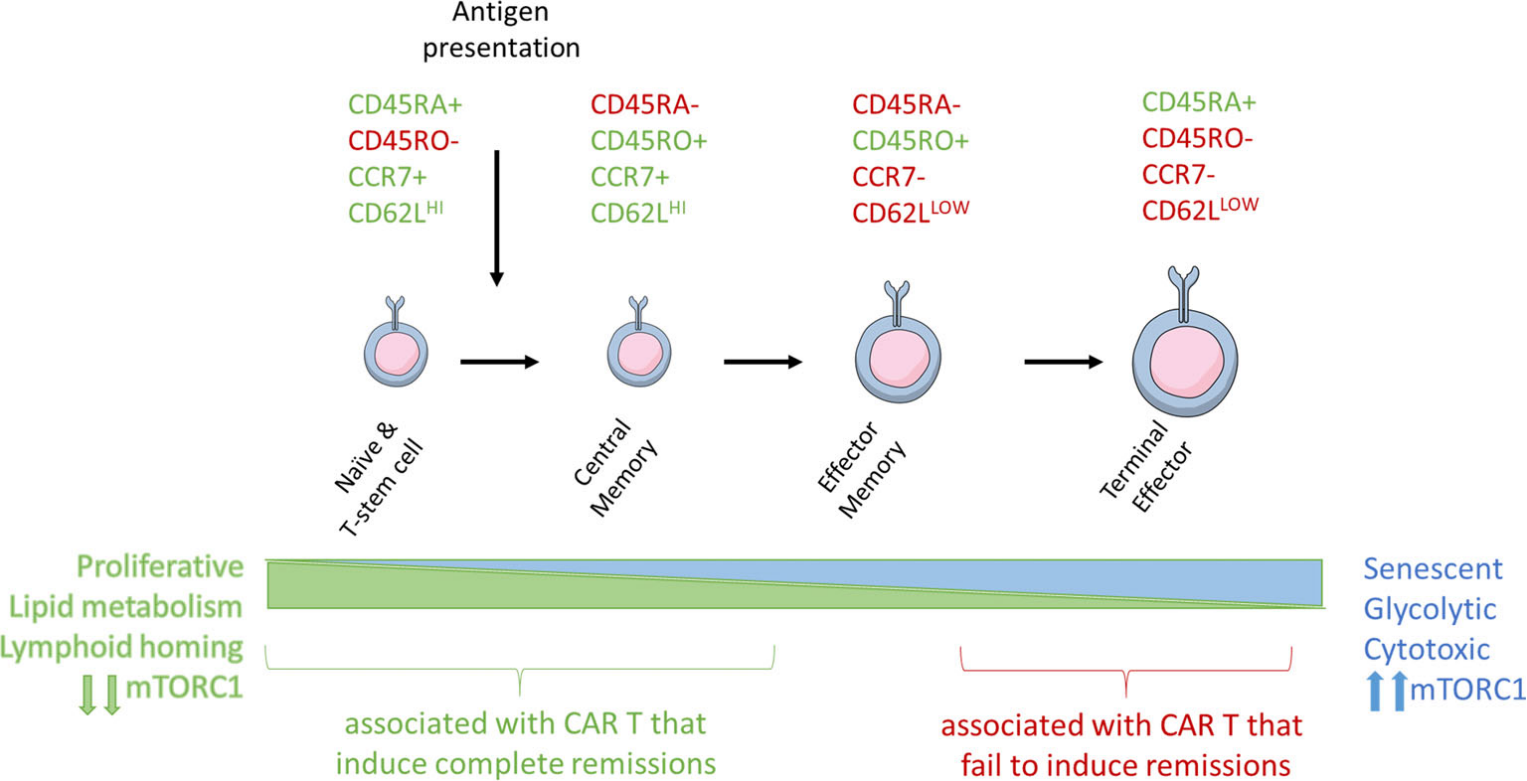
Antigen stimulation induces T-cell differentiation and associated changes in T-cell metabolism and size. Naïve, stem cell, and central memory phenotype T cells are smaller and associated with catabolic metabolism. Antigen stimulation of the TCR signals through PI3K and mTORC1 to induce metabolic reprogramming, increase in T cell size, and eventual senescence.

Upon T-cell activation, there is an increase in glucose and amino acid uptake and activation of mTORC1, which is required to maintain T-cell effector functions (43–46). Activation of mTORC1 also promotes induction and maintenance of aerobic glycolysis, which leads to increased differentiation and effector functions (46). In the context of adoptive T-cell therapies, mTORC1 mediated differentiation into effector cells render ACT less efficacious, as the survival and expansion potential of differentiated T cells is limited. Inhibition of mTORC1 activity through direct inhibition, genetic modification, inhibition of AKT, or STAT3 activation promotes formation of a pool of T-cells that are smaller in size with a stem cell memory (Tscm) and naïve (Tn) cell phenotype by inhibiting aerobic glycolysis and preventing terminal differentiation (47–50) (**Figure 2**).

The effects of PI3K activation are recapitulated clinically in APDS (Activated phosphoinositide 3-kinase delta syndrome (APDS), an autosomal dominant primary human immunodeficiency caused by heterozygous gain-of-function mutations in PIK3CD which encodes the p110δ catalytic subunit of PI3K. Similar to TCR overstimulation, increased PI3K-δ and downstream mTORC1 activity in APDS alters T-cell glucose transport, leading to unregulated differentiation and increased senescence by shifting CD8+ T-cells towards short-lived effector cells that are unable to yield memory lymphocytes (51, 52). Altered T-cell differentiation due to activating mutations in the PI3K p110δ subunit thus leads to reduced numbers of circulating reduced CCR7+ Tn and central memory (Tcm) cells and increased numbers of CD45RA−CCR7− effector memory (Tem) and CD45RA+CCR7− (Te) CD8+ T-cells. The over representation of terminally differentiated and senescent T-cells leads to lymphadenopathy, functional immunodeficiency, and increased risk for sinopulmonary infections, nodular lymphoid hyperplasia and viremia from cytomegalovirus (CMV) and/or Epstein-Barr virus (EBV) and associated B-cell malignancies (51, 52).

### T-Cell Differentiation, Exhaustion, Senescence, and Metabolism

PI3K-δ and -γ mediated activation is critical to the processes of antigen driven T-cell differentiation and induction of exhaustion, senescence, and metabolic reprogramming mechanisms (53). During *ex vivo* manufacturing of the ACT product or following chronic cancer-induced antigen presentation, constant engagement of the TCR alters T-cell phenotypes and functions, ultimately contributing to reduced cytotoxic responses.

### T-Cell Differentiation

The different T-cell subsets and their memory and effector functions have been previously reviewed in extensive detail (54). Clinically meaningful populations can be defined by patterns of expression of extracellular T cell markers including CD45RA, CD45RO, CD62L, and CCR7 (55) (**Figure 2**). As T-cells become further differentiated in response to antigen presentation, CD45RA isoform is switched to CD45RO, CCR7 is lost, and CD62L expression is reduced.

Tn and minimally differentiated Tscm cells can be phenotypically defined by their expression of the co-stimulatory molecules CD27 and CD28 in the contect of other T cell markers. Stimulation *via* CD3 and CD28 in response to a cognate antigen promotes the expansion and differentiation of Tn and Tscm T-cells into CD27+/CD28+/CD45RO+/CCR7+ Tcm cells. These cells, which have previously encountered antigen, are capable of significant proliferation and have increased activity upon antigen re-exposure, express CCR7 that mediates their homing into the peripheral tissues (56, 57). Effector-memory T-cells (Tem) retain CD45RO but lose CCR7, CD27, and CD28, which significantly reduces their homing and proliferative capacity (58). Terminal effector (Te) T-cells switch to the CD45RA+ CD45 isoform back and retain CCR7 negativity. Te cells are capable of engaging a robust cytotoxic response, but their persistence is transient, and they lack the proliferative and homing capabilities of less differentiated cells. As a consequence of the proliferative capacity of T cells at different stages of differentiation, ACT composed primarily of Te cells is predicted to have a limited persistence *in vivo* and shorter response duration.

### T-Cell Exhaustion and Senescence

Constant T-cell stimulation can also lead to onset of T-cell exhaustion or senescence, both dysfunctional T-cell states that share similar characteristics of reduced proliferation, cytotoxic activity, and metabolic capacity but have different underlying etiologies, cytokine profiles, and cell surface marker phenotypes (59). While both phenotypes often overlap in immuno-oncology, exhaustion typically occurs due to constant antigenic stimulation due to the inflammatory cancer-state, while senescence ensues when cells are forced to endure multiple, rapid signals to enterthe cell-cycle and undergo proliferative cell divisions in the face of repeated antigenic stimulation, exposure to DNA damaging agents, or other stress signals (59).

The T-cell exhaustion phenotype has been well characterized in models of chronic viral infection (60). A similar process of constant antigenic stimulation of the TCR is thought to occur within the cancer TME and contribute to establishing a population of exhausted T-cells phenotypically identified by increased expression of inhibitory receptors such as PD-1 (programmed cell death-1), CTLA-4 (cytotoxic T-lymphocyte-associated protein 4), TIM-3, LAG-3 (anti-lymphocyte activation gene-3), and VISTA (52, 61). PD-1 itself has been shown to block cell cycle progression by inhibiting CD28-mediated activation of PI3K through its immunoreceptor tyrosine-based switch motif located in the cytoplasmic tail, and its increased expression in the setting of activating PI3K mutations may be compensatory (62–64). Antibody therapies called immune checkpoint inhibitors (ICIs) targeting molecules like CTLA-4, PD-1, and its ligand, PD-L1, are now commonly used in a wide variety of cancers and will be discussed in detail further on in this review.

As a result of repeated antigen-stimulated replication and differentiation, similar to the pathophysiology seen in APDS, T-cells can become senescent and lose their ability to proliferate, manifesting less cytotoxic activity and greater cell cycle arrest (59). T-cell senescence is either replicative (telomerase-dependent) or premature (telomerase independent) (65). In replicative senescence, frequent TCR engagement leads to inactivation of the telomerase promoter, decreased telomerase expression, and activation of DNA damage signals. In aggregate, this leads to cell cycle arrest, increased expression of CD57, and loss of co-stimulatory molecules CD27 and CD28 (51, 66). On the other hand, cancer mediated T-cell senescence may be telomerase independent, and impairs T-cell stimulation *via* the TCR and reduces response rates to CAR-T therapy in conditions like CLL (67, 68). We have shown that T-cells from patients with DLBCL sorted for co-expression of CD27 and CD28 proliferate whereas T-cells lacking CD27 and CD28 are senescent (69). Further implications and mechanisms of T-cell senescence in the setting of CAR-T therapy for malignant hematologic conditions has been reviewed prior in great detail (65).

### T-Cell Metabolism

**Figure 3:**
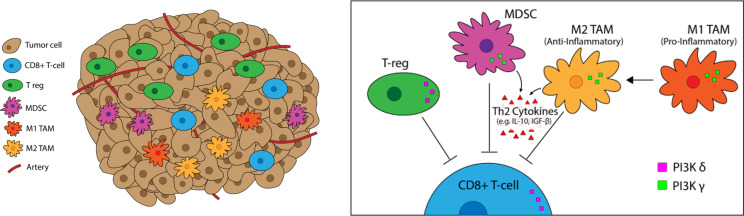
PI3K-*δ* and *-γ* in the Tumor Microenvironement. PI3K-*δ* and -*γ* signaling Tregs, TAMs, and MDSCs regulates suppression and trafficking of CD8+ tumor infiltrating lymphocytes in the TME. PI3K-*δ* signaling drives Treg suppression of CD8+ tumor-infiltrating T-cells while TAMs and MDSCs rely on PI3K*-γ* for their immunosuppressive function.

T-cell differentiation is associated with the transition from reliance on catabolic metabolism to anabolic metabolism (70). Tn, Tscm, and Tcm cells rely upon fatty acid oxidation (FAO) and oxidative metabolism (OXPHOS) to meet metabolic needs. As T-cells encounter antigen and differentiate they increase their reliance upon glycolysis to rapidly meet bioenergetic and biosynthetic demands for rapidly dividing cells, such that Te cells almost entirely rely upon glycolytic metabolism (71) (**Figure 3**). Antigen binding at the TCR upregulates glucose and amino acid transporters at the T-cell surface, driving a process of metabolic reprogramming (47, 72). During metabolic re-programming, memory cells demonstrate increases in mitochondrial mass associated with an induction of PGC1-alpha and increased respiratory capacity (73, 74). The switch to glycolytic metabolism while supporting the Te cytotoxic phenotype is also thought to reduce their longevity (75). Thus, less differentiated and less glycolytic Tn, Tscm, and Tcm cells are the preferred cell populations for ACT and other cancer immunotherapies, and strategies to abrogate metabolic reprogramming are promising mechanisms by which to improve therapy efficacy. Increased activity in the AKT/mTOR pathway drives T-cell metabolic re-programming and glycolysis, and strategies to inhibit this pathway in ACT are being tested (47–50). Changes within the tumor microenvironment also influence non-cancer cell metabolism. PD-L1 expression on cancer cells can engage with infiltrating immune effector cells that express PD-1, limiting activation of downstream mTOR signaling (72). Interestingly, PD-1 blockade has been shown to restore oxidative phosphorylation (76). Both exhaustion and senescence alter T-cell mitochondrial biogenesis and respiratory capacity, but in mechanistically different ways. In exhaustion, both OXPHOS and glycolytic metabolic mechanisms are suppressed and associated with increased PD-1 expression, while senescence is associated with a shift to towards anaerobic glycolysis (59). The significance of these changes in signaling pathways remains under investigation.

## Immune Checkpoint Inhibitors, Pi3k, And The Tumor Microenvironment

### Immune Checkpoint Inhibitor Therapy

Immune Checkpoint Inhibitors (ICI) bind to and interfere with inhibitory surface receptors on T-cells, thus allowing T-cells to remain active, participate in antigen recognition, and induce anti-tumor immune responses. Current clinical ICI applications target CTLA-4, PD-1, or PD-L1 molecules, and the FDA has approved ICI therapies in multiple malignancies, including melanoma, Merkel cell, non-small cell lung, head and neck, gastroesophageal, renal, bladder, and hepatocellular cancers (77, 78). The CTLA-4 inhibitor ipilimumab was the first ICI to be FDA approved (79, 80), and subsequently nivolumab and pembrolizumab (anti-PD-1) received approvals. Now, multiple additional ICI agents have been approved including cemiplimab (anti-PD-1) and the anti-PD-L1 antibodies avelumab, atezolizumab, and durvalumab (77). Globally, many more anti-CTLA-4, anti-PD-1, and anti-PD-L1 antibodies are under clinical study.

ICI have demonstrated tremendous clinical benefit in multiple solid tumor indications. For example, in patients with unresectable advanced stage (III/IV) cutaneous melanoma, ICIs have achieved single-agent response rates up to 40% in the first-line setting, higher than the prior 10% seen with chemotherapy alone. While these improvements in clinical outcomes are exciting, a majority of patients will not experience a long-term clinical response and not all malignancies are sensitive to T-cell checkpoint inhibitors, in large part due to poor intratumoral T-cell trafficking. A common strategy currently being used is to use a multi-targeted approach to simultaneously inhibit additional co-inhibitory receptors other than PD-1 and CTLA-4 associated with primary or acquired ICI resistance (81, 82). Promising preclinical data with co-inhibition of TIM-3 or LAG-3 with anti-PD-1 therapy (83, 84) has led to multiple ongoing clinical trials testing such combinations in solid tumor malignancies, including with nvel dual-targeting bispecific antibodies (NCT03219268, NCT04080804, NCT04140500, NCT03250832, NCT01968109, NCT04370704, NCT03005782, NCT04139902, NCT03680508, NCT02817633, NCT03708328, NCT03744468, NCT02608268, NCT03630159) (32).

An alternate strategy, however, is to combine non-ICI pharmacologic inhibitors with ICI with the intent of enhancing T-cell anti-tumor activity in ICI-resistant cancers (85, 86). Mechanistically, CTLA-4 inhibition reduces CD4+ Treg proliferation and induces the expansion of a Th1-like CD4+ effector populations, while inhibiting PD-1 reduces T-cell exhaustion and increases CD8+ tumor infiltrating subsets (87–90). ICI failure stems from fundamental deficiencies in mechanisms of innate and acquired resistance (82, 91). Variability in cancer type, prior treatment history, tumor heterogeneity, and the immunosuppressive tumor microenvironment also influence poor therapy response (92, 93). Seven immune escape mechanisms for ineffective immune mediated anti-tumor response to anti-PD-1/PD-L1 therapy have been previously described (94, 95), and can be broadly summarized into three categories, namely 1) T-cell priming and activation, 2) T-cell trafficking and infiltration, and 3) tumor cell recognition and killing.

### Immunogenic vs Immune-Restricted TME

Tumor microenvironments can be classified as *immunogenic* or *immune-restricted*, which refers to the infiltration and presence of T-cells and other antigen presenting immune cell populations (96). *Immunogenic* or ICI-responsive tumor types include cutaneous melanoma, lung cancers, renal cancers, and bladder cancers, while colorectal, pancreatic, prostatic, breast, or cancers of central nervous system origin are regarded as *immune-restricted* and ICI-resistant malignancies (97–100).

Lack of ICI response in *immune-restricted* cancers occurs from deficiencies in T-cell priming, activation, trafficking, and infiltration into the TME. Research has focused on increasing tumor antigen release, improving the presence and efficiency of antigen presenting cells, and augmenting T-cell intra-tumoral homing and co-stimulation (100, 101). In *immunogenic tumors*, ICI resistance and/or failure is more complicated and follows three distinct phenotypes: **a)** patients that do not respond (innate resistance); b) those that respond initially but fail to respond in later stages (acquired resistance); and c) those that respond initially and continue to respond (92, 93). Despite intratumoral T-cell presence, deficiencies in tumor cell recognition and killing driven by alterations in MHC or other co-inhibitory signals can lead to immune evasion and therapy failure (102–104). Even if adequate antigen presentation exists, T-cell exhaustion, senescence, and inactivation due to concomitant checkpoint pathways, or direct suppression by other immune cells including regulatory T-cells (Tregs) and M2-like tumor associated macrophages (TAMs) or MDSCs limit the efficacy of ICI. Therefore, much need exists for therapies that synergize with ICI to elicit activation, proliferation, and long-term persistence of antigen experienced tumor-reactive T-cells (105).

### PI3K-δ and -γ Effects in the TME

PI3K signaling plays a multifactorial role in shaping the TME, particularly the milieu of other T-cell suppressive immune cells (**Figure 5**). We have previously linked PI3K pathway signaling to tumor antigen presentation mechanisms in head and neck cancers. PTEN loss and PI3K activation downregulated major histocompatibility complex (MHC) Class I and Class II induction by IFN-γ, and clinical tumor samples demonstrated inverse staining associations of MHC and Phospho-S6, a serine/threonine kinase downstream of PI3K (106). Downregulation of MHC expression in HNSCC and melanoma has a clinical correlation with treatment resistance and poorer clinical outcomes (102–104). Furthermore, PTEN loss has been shown to promote resistance to T-cell targeted immunotherapy in melanoma (107). The effects of PI3K inhibition on T-cell infiltration and activation in tumors, thus leading to improved T-cell mediated cytotoxicity with ICI, has been well characterized in multiple pre-clinical models of immunogenic and immune-restricted cancers, however the mechanism of such effects depends which PI3K isoforms are targeted (108, 109). Importantly, PI3K-δ and -γ isoforms regulate lymphocyte trafficking, intratumoral lymphocyte recruitment, T-cell differentiation, activation, and proliferation, myeloid cell and macrophage differentiation and function, and immune cell metabolism (5, 109–113). Chemoattractants produced by cancers also activate GPCRs and RTKs involved in PI3K phosphorylation and activation, like PI3K-δ in immature and immunosuppressive myeloid cells, further driving and sustaining tumor inflammation (114).

#### Intratumoral T-Cell Infiltration

In cancer, PI3K-δ inhibition has been shown to play a role in augmenting intra-tumoral T-cell activation. In preclinical models of lung, breast, and colon cancer, *in vivo* treatment with the PI3K-α/δ inhibitor AZD8835 and the PI3K-δ inhibitor idelalisib favorably increased CD8+ TIL/Treg ratios by ~2-fold (36). Furthermore, *ex vivo* cultures of conventional CD8+ T-cells with AZD8835 and idelalisib demonstrated a dose-dependent enhancement in T-cell survival, cell size, increased CD69+ activation marker expression, and increased expression of the IL-2 receptor CD25 without negatively impacting proliferation (36).

In a triple negative breast cancer-like transgenic MMTV-PyMT murine model of breast cancer, an increase of more than two-fold in the percentage of intra-tumoral CD4+ and CD8+ T-cells were found in the mammary fat-pad tumors and lung metastases of PI3K-γ knockout mice versus PI3K-γ competent mice (115), along with a 50% reduction in primary tumor growth volume at 5-weeks, reduced metastases formation, increased TNF-alpha secretion by CD4+ and CD8+ TILs, and increased TIL expression of the T-cell activation marker CD69+. In PI3K-γ competent mice, inhibition with the pan-PI3K inhibitor buparlisib recapitulated the above findings. The most compelling finding is that while single-agent anti-PD-1 antibody therapy is minimally effective in this TNBC model, combination therapy of anti-PD-1 antibody with buparlisib inhibited tumor growth in 100% of the mice. Similar findings were also seen in syngeneic mouse models of pancreatic ductal cancer, with tumors from PI3K-γ knockout mice exhibiting significantly more CD4+ and CD8+ T-cell content than tumors from wild-type mice (116). The most likely explanation for these observations is that PI3K-δ blockade inhibits Tregs and PI3K-γ inhibition suppresses MDSCs and TAMs, thereby indirectly and favorably altering the T-cell immune infiltrate (3, 6, 109, 117).

#### Regulatory T-Cells (Tregs)

Tregs represent a diverse and functionally distinct population of T-cells involved in immunological self-tolerance. Deficiencies in Treg function can lead to clinical manifestations of autoimmune disease while increased Treg presence in cancer enhances immune escape by inducing poor CD8+ T-cell tumor infiltration and cytotoxic activity (118). A commonly accepted Treg phenotype is CD4+CD25+Foxp3+CD127lo, and the critical role of PI3K-δ in maintaining Treg proliferation and immunosuppressive function was initially demonstrated in mouse models of colitis (119). Specific to cancer immunology, work in murine models has shown PI3K-δ knockout impairs Treg proliferation and redundancy in the PI3K-α and -β pathways in conventional T-cells spares their function in the face of pharmacologic PI3K-δ inhibition. Similar results have been seen with pharmacologic inhibition in human T-cell populations as well (3, 35). PI3K-δ inhibition with AZD8835 (PI3K-α/δ) and PI-3065 (PI3K-δ specific) decreased tumor Treg infiltration by over 50% as early as 3 days after treatment in the CT26 colorectal mouse model (36). Similar results of increased tumor reduction and reduced numbers of Tregs, M2-TAMs, and MDSCs were seen in A20 lymphoma and CT26 colorectal mouse models with combination duvelisib and anti-PD-1 antibody therapy versus anti-PD-1 therapy alone (120).

Characterizing Treg in blood samples from patients with CLL prior to and on treatment with idelalisib suggest that idelalisib therapy diminished Treg immunosuppressive activity (121). In patients with advanced melanoma, increased numbers of Tregs have been seen in the peripheral blood and tumors (primary, lymph nodes, and metastatic sites) of patients (15). Overall, large meta-analysis from clinical studies have shown that increased Treg infiltration is associated with reduced OS in a majority of solid tumors, including cervical, renal, melanomas, and breast cancers (122).

#### TAMCs (Tumor Associated Myeloid Cells)

TAMCs encompass a wide phenotype of myeloid-derived cells that include MDSCs and TAMs. Our current understanding of myeloid cell biology suggests that cancers generate pro-inflammatory factors that disrupt normal bone marrow myelopoiesis, increase the expression of immunosuppressive factors like arginase and inducible nitric oxide synthase, and lead to the expansion of a heterogenous population of immunosuppressive immature myeloid cells (IMCs), also known as MDSCs (123, 124). While variability in the literature exists, MDSCs are phenotypically accepted to be CD33+CD11b+HLA-DR-/low in humans and CD11b+Gr1+ phenotype in mice (123, 125), and can be further divided according to either a granulocytic or monocytic (mMDSC) phenotype (126). In humans, CD14+HLA-DR-/low and CCR2+ mMDSCs are regarded as the most immunosuppressive subtype (127–129). TAMs comprise a heterogenous population of CD45+ cells residing within the TME that originate from normal tissue precursors or circulating myelomonocytic progenitors (130). TAMs are often characterized based on the classically accepted pro-inflammatory M1- or anti-inflammatory M2- macrophage phenotypes, named after the respective Th1 and Th2 cytokines with which their responses are associated. While MDSCs and TAMs are often simplified as M2-like (M2*) and characterized by high levels of IL-10 secretion (131, 132), TAM diversity is complex and unlikely yet fully elucidated, but we surmise that the majority of TAMs functionally fall on a spectrum somewhere in-between (133–136). The complex myelomonocytic checkpoints governing the differentiation of TAMs from myelomonocytic progenitors can broadly be considered as 3 steps: **1)** IMC expansion and differentiation into MDSCs, **2)** MDSC migration and differentiation into TAMs, and **3)** TAM polarization, with each step being mediated by growth factors (GM-CSF (granulocyte macrophage colony stimulating factor); G-CSF), cytokines (IL-10, IL-6) and chemokines (CCL2, CCR5) released either by the tumor cells or the surrounding stroma (137).

Within the TME, analysis of the myeloid infiltrate with PI3K-γ knockout or inhibition demonstrates an increase in M1/M2 TAM ratios (109, 116). Findings are similar with the dual PI3K-δ/γ inhibitor duvelisib in PDX models of T-cell lymphoma, in which duvelisib reduces the percentage of M2-phenotype macrophages in the mouse spleens with a concomitant 50% increase in M1-phenotype macrophages (138). In PI3K-γ murine knockout models, intratumoral migration of TAMs and their immunosuppressive activity (as measured by cytokine production) is severely impaired in the knockout versus wild-type mice, demonstrating the critical, non-redundant role PI3K-γ plays in myeloid cell activity (6, 115, 116). PI3K-γ expression in myeloid cells in murine pancreatic cancer models is associated with transcription of genes associated with the M2-macrophage phenotype in pancreatic cancer, including immunosuppressive factors like Arg1, TGF-beta, and IL-10. Inhibiting PI3K-γ induced expression of these genes permits for CD8+ T-cell activation and reduced cancer survival (116).

De Henau et al. demonstrated that combination anti-PD-1 and anti-CTLA-4 therapy had minimal tumor growth impact *in vivo* in mouse models of 4T1 (breast) and B16-GMCSF (melanoma), but that adding the PI3K-γ selective inhibitor eganelisib to the combination therapy resulted in 30% and 80% complete remissions in each model, respectively (109). Dual PI3K-δ/γ inhibition, in combination with PD-1 pathway inhibition, resulted in MDSC inhibition and increased CD8+ T-cell infiltration in preclinical models of HNSCC and osteosarcoma (108, 139). These findings are further supported by the fact that selective PI3K-γ inhibition had minimal impact in a B16F10 melanoma model, presumably due to lower numbers of baseline suppressive TAMCs, in contrast to results seen in the GMCSF expressing B16-GMCSF model. Clinically, ICI resistance has been well characterized to correlate with the presence of TAMCs, as increased circulating levels of MDSCs have been shown to correspond with poor response to anti-CTLA-4 therapy in melanoma patients (140).

## Adoptive Cell Transfer Therapies

### Overview of ACT

CART and TIL therapies use similar fundamental steps of harvesting T-cells, *ex vivo* manufacturing, lymphodepletion therapy, and infusion of final T-cell product. T-cells can be harvested from peripheral blood *via* apheresis for CART and TCR or isolated from tumor for TIL therapy. For CART therapy, the T harvested cells are transduced or transfected with genetic material encoding a new synthetic TCR (T-cell receptor), called a chimeric antigen receptor (CAR), which is able to bind to a specific antigen expressed on the surface of tumor cells. In contrast to ICIs, no ACT therapies are currently approved to be used in the first-line treatment setting.

The majority of efforts in CART therapy have focused on targeting CD19 in B-cell mediated hematologic malignancies. Clinically, CART is reserved for patients who have disease refractory to multiple prior lines of therapy with limited third-line options and poor prognosis. In DLBCL, for example, approximately 50% of patients with relapsed or refractory disease will progress following stem cell transplant, and median survival is ~6 months without any additional treatment, like anti-CD19-CART (141).

Four commercially available FDA approved anti-CD19-CART therapies currently exist. Kymriah (tisagenlecleucel) is the only CART approved for two distinct indications: adults with relapsed or refractory DLBCL, high-grade B cell lymphoma, and DLBCL arising from follicular lymphoma (142) and young adults up to age 25 years of age with relapsed or refractory B cell acute lymphoblastic leukemia (B-ALL) (143). Axicabtagene lisoleucel (Axi-cel; Yescarta) is also approved for adult patients with relapsed or refractory large B cell lymphomas, including DLBCL, primary mediastinal B cell lymphoma, high grade B cell lymphoma, and DLBCL from transformed follicular lymphoma based on results from the ZUMA-1 trial (144). The similarly approved lisocabtagene maraleucel (liso-cell; Breyanzi) is the only CART product in which CD4+ and CD8+ CART are separately manufactured and administered as sequential components in equal doses (145). Approved for the treatment of adult patients with relapsed/refractory mantle cell lymphoma (MCL) is brexucabtagene autoleucel (brex-cel; Tecartus; KTE-X19), a CD19 CART construct similar to axi-cel (146). KTE-X19 differs in that manufacturing incorporates a process of T-cell selection and lymphocyte enrichment to prevent contamination of the CART product with circulating mantle cell lymphoma cells that could contribute to disease relapse. The most recently approved CART is idecabtagene vicleucel (ide-cell; Abecma), which targets BCMA (B cell maturation antigen), a TNF family cell surface receptor commonly expressed in multiple myeloma, for the treatment of patients with multiple myeloma refractory to or relapsed after at least three prior treatments (147, 148). Another anti-BCMA CART therapy, JNJ-4528, has received breakthrough designation by the FDA based on results from the CARTITUDE-1 trial for patients with relapsed/refractory multiple myeloma (149, 150).

In contrast to CART, in TCR therapy, the T-cells are transduced with an engineered-HLA specific TCR that binds to MHC on cancer cells, can target a wider variety of cancer antigens, and is more sensitive than engineered CART (151). Unlike CART, however, no TCR therapies are currently approved for clinical use, but multiple agents are in clinical trials with a particular interest in targeting the cancer-testis family antigen NY-ESO-1 (New York esophageal squamous cell carcinoma 1). The clinical development of TCR therapies, including common indications and antigen targets, have been excellently reviewed (152). For the purposes of this review, both technologies face similar challenges with respect T-cell harvesting, *ex vivo* expansion, final product infusion, and clinical toxicity, and in this context, we will primarily focus on CART in the context of clinical challenges and limitations.

In contrast to T-cell harvesting from peripheral blood, TIL therapy harnesses the power of a patient’s own tumor fighting T-cells by isolating them from autologous tumor tissue (isolation step), growing and activating those cells *ex vivo* (expansion step), treatment with lymphodepleting chemotherapy prior to TIL infusion (pre-conditioning step), infusing expanded TILs, and supporting *in vivo* T-cell activation and proliferation post TIL infusion with high-dose interleukin-2 therapy (HD-IL-2) (153). While no FDA approved TIL therapies currently exist, promising options are on the horizon (154). The history of research work on TIL therapy over the past 30 years and recent advancements in treating melanoma and other cancers with TIL has been excellently reviewed previously (155–157). Initial studies were performed in patients with metastatic melanoma in the age prior to ICI when therapy options were limited (153), and since then TIL therapy has been further refined and clinically explored in other epithelial cancers, including ovarian, cervical, gastrointestinal and renal cell (156, 158–161). TIL therapy requires patients must have (1) a resectable tumor from which (2) TILs can be isolated that must (3) exhibit tumor-specific reactivity. *Ex vivo* TIL expansion is a resource intensive process that traditionally takes 5-8 weeks (162).

### ACT Product Limitations

All ACT therapies require engaging the antigen receptors to promote *ex vivo* expansion to produce enough manufactured product and *in vivo* expansion and persistence to generate and maintain therapeutic response. Many pitfalls to the manufacturing process exist, including the challenges of sustained antigen stimulation that promotes T-cell differentiation, exhaustion, and eventually senescence (65, 163). Cancer and its inherent chronic inflammatory state can further impair T-cell mitochondrial function and metabolic fitness, rendering ACT less effective (67). Current strategies designed to promote formation of less differentiated cells and improve metabolic and cytotoxic capacity involve synergistically combining ACT with other treatments, like anti-PD-1 therapy and anti-OX40 antibody therapy, or directly modifying CART to express costimulatory receptors (CD27), stimulatory cytokines, or ICIs (“Armored CAR”) (164). Adding additional agents to the *ex vivo* T-cell expansion process to improve T-cell selection and expansion are also under consideration, including Bruton’s tyrosine kinase (BTK) inhibitors and homeostatic cytokines like IL-7, IL-15, and IL-21 to promote the memory stem cell phenotype (165, 166).

**Figure 4:**
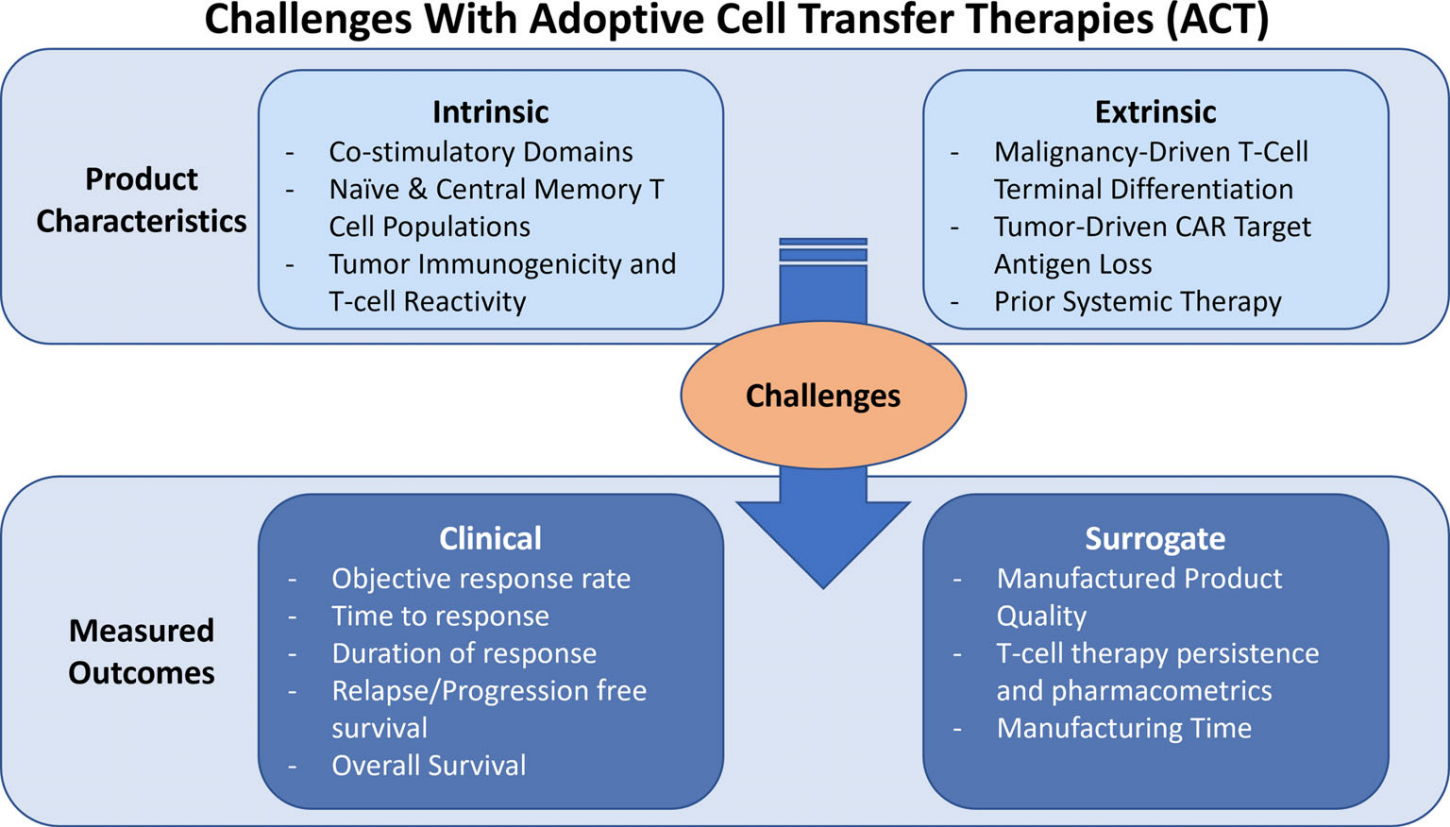
Addressing Challenges Associated with Intrinsic and Extrinsic Product Characteristics in ACT. Challenges with ACT pertain to intrinsic and extrinsic characteristics that limit success of the manufactured product. Intrinsic characteristics are T cell specific and extrinsic include cancer-induced pressures or prior therapy effects. Measured outcomes can be considered as clinical or surrogate outcomes.

Both the T-cell intrinsic properties of the ACT product and extrinsic factors related to the patient clinical history and tumor microenvironment (TME) may promote or limit the clinical efficacy of ACT (**Figure 4**). Intrinsic characteristics pertain to either the CART construct or the phenotypes of T-cell subsets in the ACT product, while extrinsic characteristics relate to cancer biology or prior therapy induced negative effects on T-cells. The impact of these factors directly augment or inhibit the success of the manufacturing process and long-term efficacy of the transferred cells.

#### Intrinsic Characteristics That Limit ACT Success

##### Co-Stimulatory Domains

Functional and phenotypic differences between the manufactured products are a consequence of heterogeneity between CART constructs, patient demographics, and the manufacturing process. These factors, taken together with the heterogeneity of patients across difference clinical trial designs, make direct comparisons of the efficacy of competitor products and trial outcomes challenging. All FDA-approved therapies are second generation CARTs and contain both a TCR stimulatory domain (from the T-cell surface glyco-protein CD3 ζ-chain (CD3ζ) and a co-stimulatory domain (167). Co-stimulatory domains of currently approved CART are either CD28 or 41BB (CD137). Axicabtagene ciloleucel and Brexucabtagene autoleucel are CD28-based, while tisagenlecleucel (CD19), lisocabtagene maraleucel (CD19), and idecabtagene vicleucel (BCMA) utilize 41BB. Data comparing the effect of these co-stimulatory domains, while predominantly preclinical or from small-scale clinical investigations, suggest that the type of co-stimulatory molecule expressed may contribute to differences in expansion kinetics, persistence, and efficacy amongst approved CART products (168–171).

41BB co-stimulation appears to induce growth of CD8+ Tcm cells with increased respiratory capacity, fatty acid oxidation, and mitochondrial biogenesis, while CD28 co-stimulation induces Tem with augmented glycolytic activity (171). The reason for these differences appears to lie within the distinct signaling pathways each co-stimulatory domain activates (172). In pre-clinical models, 41BB-CD19-CART demonstrated increased gene transcription of transcription factors associated with memory function (KLF6, JUN, JUNB), while similar CD28-based CART showed increased surface markers of exhaustion (TIM-3, LAG-3, CTLA-4). These differences in gene expression and enrichment pathways suggest 41BB signaling may prevent T-cell exhaustion, improving the persistence of T-cells *in vivo* following treatment of leukemic mice. That said, a limitation of 41BB-CART is the inability to rapidly proliferate to control tumor burden (173). In contrast, CD28-CART proliferate more rapidly than 41BB-CART but fail to persist long-term. Taken together, the use of each co-stimulatory molecule in the CART construct contributes distinct beneficial properties, with CD28-CART possessing a greater proportion of Tem cells capable of eliciting a rapid short-term effector memory response and 41BB-CART having more Tcm cells that induce long-term effector T-cell (Te) function with increased persistence (167, 173). Not surprisingly, development of third generation CART constructs that encode both CD28 and 41BB is underway (174).

##### Naïve and CM T-Cell Phenotype

T-cell differentiation has broad implications on proliferative capacity, effector function, and metabolic reprogramming. The intrinsic phenotypic differences, existing either pre- or post-manufacturing, thereby significantly influence quality of the manufactured product and subsequent therapeutic success.

Recent work by Fraietta et al. in a study of 41 patients with relapsed and refractory CLL having received anti-CD19-CART retrospectively associated complete remissions following CART therapy with an increased frequency of a class of memory-like CD8+CD27+CD45RO- T-cells in the leukapheresis sample prior to CART manufacture based on cluster analysis (68). Similarly, complete remissions following CART therapy were more likely in patients receiving products containing reduced frequencies of senescent CD8+PD-1+ CART cells (20% vs 50% or higher in PR/non-responders) and increased frequencies of Tcm-like CD8+CD27+PD-1- CART cells. The improved disease remission rate seen with the CD27+ memory like T-cells is anticipated to have a direct effect on *in vivo* expansion and subsequently long-term CART persistence. Clinical trial data with axi-cel (axicabtagene ciloleucel) demonstrated that higher CART levels in peripheral blood in the first 4 weeks following treatment were associated with increased treatment response and that detection of CART in the peripheral blood up to 2 years correlated with long-term ORRs (objective response rates) (144). Conversely, we have reported that oligoclonal expansion of CD27/CD28 double-negative T-cells in response to pancytopenic aplasia, led to rapid disease relapse in a patient following CART therapy (175).

Taken together, optimization of CAR constructs and a greater understanding of favorable CART T-cell phenotypes on intrinsic function are areas for further research. Unfortunately, standard methods for CART manufacturing at this time do not automatically enrich the product for favorable T-cell phenotypes and inadvertently may also promote antigen-driven terminal differentiation (41, 176–178).

##### Tumor Immunogenicity and T-cell Reactivity

TIL products rely on the intra-tumoral presence and isolation of tumor antigen specific T-cells. In less immunogenic malignancies or malignancies that lack high acquired/somatic mutational load, expanding tumor reactive T-cells has been challenging. In recent years, this process has benefited from newer high-throughput genetic sequencing technologies that can identify tumor-specific mutations and neoantigens that can be targeted by TILs. Advances in whole exome sequencing have enabled investigators to selectively isolate and expand tumor and peripheral blood T-cells reactive against those tumor epitopes for TIL therapy (179). Such advancements allow researchers to identify patient specific, targetable, and somatic mutations in epithelial cancers like breast (BC), esophageal, and ovarian cancers and employ them in TIL therapy (180). A recent study of a patient with chemo-refractory metastatic HR+ (Hormone Receptor) BC with a complete response following treatment with autologous TILs demonstrated the infused product was reactive against mutant versions of 4 different proteins (SLC3A2, KIAA0368, CADPS2, CTSB) (181). Other approaches include selecting TILs from the expanded product that demonstrate a specific phenotype, such as with LN-145-S1, a PD-1 selected TIL therapy under investigation in head and neck cancers (Clinicaltrials.gov Identifier: NCT03645928) (32).

#### Extrinsic Characteristics That Limit ACT Success

##### Malignancy-Driven T-Cell Terminal Differentiation

Patients with hematologic malignancies, especially those having had multiple lines of cytotoxic chemotherapy, have more senescent T-cells (67, 68) and fewer of the memory-like CD8+ cells that are associated with durable remissions after CART therapy. The level of cancer induced T-cell senescence also varies by cancer-type, especially among chronic versus acute CD19+ B cell malignancies. In CLL, there is a T-cell-intrinsic disadvantage that exists prior to initiating CART manufacturing that is likely related to CLL-induced immune dysfunction (67, 68). These T-cell intrinsic defects may potentially explain the finding that meta-analysis of clinical trials shows lower rates of CD19-CART-mediated remissions in CLL compared to other hematologic malignancies (2, 182).

##### Tumor-Driven CAR Target Antigen Loss

Disease relapse after CD19-CART therapy can be antigen positive (CD19+) or antigen negative (CD19-). 10-20% of patients with CD19+ malignancies can develop native-antigen negative disease (CD19 mutation or down regulation) that is challenging to treat or re-treat with CD19 CART (183). Approaches to overcome antigen loss include designing CAR constructs that target multiple antigens, such as CD19 and CD22 for ALL (Acute Lymphoblastic Leukemia) (NCT03241940 and NCT03289455), ALL and diffuse large B cell lymphoma (DLBCL) (NCT03233854), ALL and non-Hodgkin lymphoma (NHL) (NCT03330691 and NCT03448393), and NHL and CLL (NCT03019055). With dual-recognition, CARTs can engage either antigen and tumor cells must lose expression of both antigens concomitantly for escape.

Clinically, disease relapse due to antigen loss or lack of antigen presence is less common than antigen positive relapse, which more often is related to lack of CART persistence, low CART potency, or B cell aplasia (183). In the Phase II JULIET trial with tisagenlecleucel in patients with DLBCL, no differences in response between groups stratified by tumor expression of CD19 were seen (142). With axi-cel, further analysis of CD19 expression at the time of progression showed that only 3 (27%) out of 11 patients with CD19+ baseline disease had CD19- disease at progression (144).

The sensitivity of methods used to assess epitope presence also matters. In the axi-cel trial, 8 patients with assay-tested CD19- disease were included in the trial, and response rates in these 8 patients were similar to trial participants with assay-confirmed CD19+ disease. While not powered to specifically examine this, the positive responses to anti-CD19 CART even with epitope negative disease suggests that current antigen detection assays may not entirely or accurately assess target antigen presence.

##### Prior Systemic Therapy

Most FDA approved indications for CART include patients with relapsed or refractory aggressive B cell lymphomas, and therapy for such patients often involve high dose chemotherapy with stem cell transplantation. An unintended consequence of cytotoxic chemotherapy is the depletion of Tn, Tscm, and Tcm subsets that possess the greatest expansion potential and anti-cancer activity (184, 185). DLBCL patients with a history of extensive prior chemotherapy have fewer naïve and minimally differentiated T-cells, including increased populations of CD27 and CD28 double- negative senescent cells, when compared to healthy controls and newly diagnosed DLBCL patients (69).

Lymphodepletion with chemotherapy prior to ACT infusion also impacts clinical efficacy. In patients with NHL undergoing CD19 CART, patients who received combination cyclophosphamide with fludarabine versus cyclophosphamide alone had increased CART expansion, persistence, and objective response rates, especially CRs (50% vs 8%) (186). Lymphodepletion has broad benefits, including killing of immunosuppressive regulatory T-cells (Tregs) and myeloid derived suppressor cells (MDSCs), elimination of T-cells with cytokine receptors that function as homeostatic cytokine sinks resulting in increased levels of cytokines like IL-7, IL-15, and IL-21 necessary for *in vivo* ACT expansion (187), and direct modulation of tumor IDO (indoleamine 2,3-dioxygenase) (188), all of which together beneficially support T-cell persistence and cytotoxic activity (189). In preclinical models, TBI (Total Body Irradiation) has been shown to increase microbial Toll-Like Receptors (TLRs) that activate antigen presenting cells (190). The impact of prior systemic therapies on CART success suggests there is potential for treatment with additional immunomodulatory therapies that favorably alter *in vivo* frequencies of Tn and Tcm cells prior to T-cell isolation or suppress Tregs and other immunosuppressive cell populations and alter circulating cytokine levels prior to CART infusion.

### Measured Outcomes With ACT

#### Clinical Outcomes and Toxicities

##### CART Therapy Outcomes

Clinical outcomes with CD19 CART vary based on the B-cell malignancy histology, patient demographics, and intrinsic features of the manufactured product, including co-stimulatory domains engaged and CD4/CD8 T-cell ratios. Meta-analysis of 42 trials of anti-CD19 CAR T-cells in various B cell hematologic malignancies showed higher complete response (CR) rates of 77% in acute lymphoblastic leukemia patients (ALL) versus 25% in chronic lymphocytic leukemia (CLL) and 54% in non-Hodgkin lymphoma (NHL) (2). Across all four commercially available CD19 CART therapy approval trials, except for B-cell ALL, the primary end points were ORR (CR or PR (partial response)), with additional metrics of time to response, duration of response (DOR), relapse free survival (RFS), overall survival, and CART kinetics also investigated. Clinical response rates (CR and PR) from pertinent clinical trials of the above CART therapies are reviewed in detail in **Table 1**.

**Table 1:** Clinical Outcomes from CART FDA Approval Trials.

CART Trial	CART Therapy	Phase	Indication	Minimum # of Prior Therapies	ORR (CR/PR)
JULIET	Tisagenlecleucel	Phase II	R/R DLBCL post or ineligible for ASCT	2	52% (40%/12%)
ELIANA*	Tisagenlecleucel	Phase II	R/R B-ALL (<25 yo)	1	81%* (-/-)
ZUMA-1	Axi-cel	Phase II	R/R DLBCL, PMBL, or TFL	2	82% (54%/28%)
Transcend NHL 001	Liso-cel	Phase I	R/R DLBCL, FL, PMBL	2	73% (53%/20%)
ZUMA-2	KTE-X19	Phase II	R/R MCL	3	85% (59%/26%)
KarMMA	Ide-Cel	Phase II	R/R Multiple Myeloma	3	73% (33%/40%)

ORR (objective response rate), CR (complete response rate), PR (partial response rate), R/R (relapsed/refractory), ASCT (autologous stem cell transplant), PMBL (Primary Mediastinal B cell lymphoma), FL (Follicular Lymphoma), TFL (transformed Follicular Lymphoma), MCL (Mantle Cell Lymphoma).

Currently approved CART therapies by trial name, indication, and clinical response rates.

*Prior therapy could include autologous stem cell transplant, ORR, Overall remission rate.

Taking these findings together, CART response rates in high grade B-cell lymphomas are generally >50% regardless of the therapy given. Of note, the median time to clinical response appears to vary according to clinical trial-specified assessments (1 vs 3 months), and in some cases, delayed CRs have been observed. In Zuma-1, the first tumor assessment was at 1-month, which corresponded with the median time to response, however 23 patients (11 of 35 with a partial response and 12 of 25 with stable disease) developed CRs in the absence of additional therapies up to 15 months after CART. The heterogeneity in patient populations and prior therapy received between trials, variability in observed CR and PR rates with similarly designed CART products, and observation of delayed ORRs beyond pre-determined clinical timepoints suggest we still have much learn about how we assess clinical outcomes with CART.

##### CART Therapy Toxicity

Toxicity following CART infusion is common, with the most worrisome being CRS (cytokine release syndrome) and neurotoxicity (2). In CRS, infusion of cytotoxic T-cells leads to a rampant release of cytokines like IFN-γ, GM-CSF, TNF, IL-10, and IL-6, triggering an inflammatory response characterized by tachycardia, risk for acute respiratory distress syndrome (ARDS) acute hypoxic respiratory failure, and multiorgan failure (191). CRS is treated by administering the IL-6 inhibitor tocilizumab or steroids, both of which can reduce the morbidity and mortality associated with CRS. Tocilizumab does not appear to suppress the cytotoxic activity of the infused T-cells, and is generally preferred to steroids as initial therapy (192). Neurotoxicity from CART is biologically less well understood, but symptomatically recognizable. Neurotoxicity can occur with CRS, after CRS, or in delayed form multiple weeks after infusion, has been observed occurring in all grades as high as 64% (axi-cel). Across the aforementioned approval trials, Grade 3 & 4 CRS rates were highest with tisagenlecleucel (DLBCL: 22% and ALL: 46%) and lower for axi-cel (13%), liso-cel (2%), KTE-X19 (15%), and ide-cel (5%). The most common other Grade 3 or higher adverse event with CART is cytopenia, with neutropenic predominance. In the KTE-X19 and ide-cel trials, more than 90% of patients experienced Grade 3 or higher cytopenias. Direct comparisons between individual CART product toxicities are challenging due to differences in the grading criteria used between trials.

##### Current TIL Therapy Outcomes

TIL therapy has shown promise as an effective cancer therapy. Results from 93 patients with metastatic melanoma receiving lymphodepletion and TIL infusion demonstrated an ORR of >50% with 22% experiencing (n=20) complete tumor regression. At 3 years, 19/20 were still in complete response (193). In comparison, single agent HD-IL-2 therapy, which is FDA approved in this therapy refractory setting, has a 5-10% ORR (194). In a smaller study of 9 patients with recurrent metastatic cervical cancer, 3 patients had an ORR with 2/3 being CRs (159). Studies in ovarian cancer, kidney, gastrointestinal, and head and neck cancers have also been attempted, with mixed results (156, 158, 195). A critical challenge to assessing the clinical value of these data in today’s clinical context is that many past studies with TIL therapy occurred prior to the development of ICI therapies like anti-PD-1/PD-L1 and anti-CTLA-4.

Recent advancements in commercial TIL production have improved upon the initial limitations of TIL therapy, and recent clinical trial data strongly suggests this modality will become part of the clinical paradigm as an option after current standard of care in the near future. LN-144 (lifileucel) is a commercially produced TIL therapy that has a TIL manufacturing time of 22 days (Gen-2 TIL) and has demonstrated clinical benefit in cancers like melanoma, cervical cancer, and head and neck cancers. In a Phase 2 clinical trial (NCT02360579), LN-144 demonstrated a 38% (n=18) ORR (1 CR, 17 PR) in 47 metastatic melanoma patients previously treated with anti- PD-1 antibody and/or BRAF inhibitor (DOR 1.3-14.0 months) (154). Similar results were seen in 13 recurrent metastatic squamous cell carcinomas of the head and neck cancer patients (31% ORR) (NCT03083873) (196) and in 27 recurrent, metastatic or persistent cervical carcinoma patients (44% ORR, 11% CR, 33% PR) treated with lifiluecel LN-145 TIL therapy (NCT03108495) (197). LN-145 trials are ongoing in patients with metastatic triple negative BC (NCT04111510) and other bone and soft tissue sarcomas (NCT03449108) (32).

##### TIL Toxicity

TIL therapy toxicity lacks the CRS and neurotoxicity seen with CAR-T therapy, is most often related to the consequences of the pre-conditioning non-myeloablative therapy or post-infusion HD-IL-2 therapy, and is generally short lived (<2 weeks).

In trials with commercially produced TILs (NCT02360579, cohort 2, n=47), the most frequently reported toxicities of any grade (>50% of patients) were thrombocytopenia, chills, neutropenia, febrile neutropenia, anemia, and pyrexia. Approximately 95% of patients reported Grade 3 & 4 toxicities, with the most common being thrombocytopenia (81%), neutropenia (53%), febrile neutropenia (53%), anemia (47%), and leukopenia (43%) (154). Similar results were seen in cervical cancer (NCT03108495) (n=27), with Grade 3 & 4 toxicities observed in 96% of patients, the most frequent being anemia (56%), thrombocytopenia (44%), neutropenia (30%) and febrile neutropenia (30%) (197). Myeloablative regimens, typically combining chemotherapy with TBI, may improve ORRs and CRs, albeit with increased clinical toxicity. NCT01319565 is a clinical trial evaluating the addition of TBI to pre-conditioning regimens prior to TIL therapy (32).

#### Surrogate Outcomes

While there is much interest in developing surrogate endpoints to determine ACT success, no standardized metrics currently exist. However, surrogate outcomes like manufacturing success and ACT cell product persistence *in vivo* may become valuable metrics in future clinical trials.

##### Manufactured Product Quality

Failure to manufacture or complications while awaiting CART therapy are measurable outcomes that indirectly affect planned CART therapy for patients with aggressive disease. While most patients that undergo leukapheresis are able to receive CART therapy, manufacturing failure remains a limitation in the success of this novel therapy. Reasons contributing to manufacturing failure include the collection of insufficient numbers of T-cells from pre-treated patients or contamination of the apheresis product with granulocytes and monocytes (198). Other quality control issues related to post-manufacturing viability, product purity, or product potency, as determined by quantitative or qualitative cytotoxic or cytokine release assays, may also render the final product unsuitable for infusion (198). Early non-commercial CART products had higher manufacturing failure rates from 2% as high as 14%. In the post-marketing-approval use of CART, rates of manufacturing failure have continually decreased (**Table 2**).

**Table 2:** Clinical Experience With CART Manufacturing.

CART Therapy	Number Enrolled	Manufacturing Failure % (#)	Manufactured & Not Received % (#)	Total Not Received % (#)
Tisagenlecleucel	165	7% (12)	23% (38)	30% (50)
Tisagenlecleucel (B-ALL)	92	8% (7)	11% (10)	18% (17)
Axi-cel	111	1% (1)	8% (9)	9% (10)
Liso-cel	344	<1% (2)*	14% (48)	15% (50)
KTE-X19	74	4% (3)	4% (3)	8% (3)
Ide-Cel	140	<1% (1)	8% (11)	9% (12)

Rates for failure to receive therapy due to progression of disease or other complications from time of pheresis to CART infusion are shown. Total failure to receive therapy rates range from 8% to 30%. (*8% (25/344) received a non-conforming CART product not meeting criteria for liso-cel).

Manufacturing failure rates in clinical trials for FDA approved therapies range from <1% to ~8%*.

##### T-Cell Therapy Persistence and Pharmacometrics

Long-term CART persistence correlates with response rates. In the Zuma-1 Phase II trial, CART cells peaked in the blood within 14 days post infusion and were detectable up to 180 days for most patients. At 24 months, 3 patients with CRs still had detectable peripheral blood CART transgene levels (144). Similarly, with tisagenlecleucel, CAR transgene levels were measurable in the peripheral blood up to 2 years in patients with durable responses (142). Kinetic studies with BCMA CART showed 29 of 49 patients (59%) had cells present at 6 months and 4 of 11 patients (36%) at 12 months post infusion (148). Delayed CRs, especially those occurring 3 months or more after therapy infusion, raise important questions about CART pharmacometrics and time to optimal response. In Zuma-2, the median time to CR was 3.0 months but ranged from 0.9 up to 9.3 months (146). Even more impressive, delayed CRs reported in ZUMA-1 were seen up to 15 months in the absence of additional therapies (144).

Multiple strategies to improve CART persistence have been considered. Controlling the ratio of CD4 and CD8 T-cells in the infused product and using lymphodepleting fludarabine chemotherapy prior to infusion are some promising methods (199, 200). Additional strategies being considered include building newer generation CARs with multiple costimulatory domains in addition to CD28 or 41BB, like ICOS, or modifications in CD28 amino acid residues (201, 202). Reduction in target CAR antigen levels, either in direct response to CART therapy or through cancer-mediated downregulation results in loss of CAR stimulation and CART persistence. Strategies involving maintenance therapy that bolsters target antigen levels have demonstrated promise in preclinical models (203).

##### Manufacturing Time

Manufacturing time indirectly affects CART efficacy. Patients awaiting CART therapy have aggressive disease and are at high risk of complications by cancer, cancer progression, or other co-morbidities. Adverse events while awaiting CART inevitably renders a portion of patients ineligible to receive the manufactured product (**Table 2**). Typical manufacturing times range from 2-4 weeks, with the median times from leukapheresis to product delivery for axi-cel and KTE-X19 reported as 17 and 16 days, respectively (144, 146). For JULIET, 38 (23%) of 165 enrolled patients discontinued study participation for reasons unrelated to manufacturing, of which 34 of 38 were related to disease progression (142). In the tisagenlecleucel ALL trial, ~11% (10/92) of patients died prior to receiving CART due to disease progression (n=4), complications from sepsis, respiratory failure and fungemia (n=3), and other complications (n=3) (143). In the ZUMA-1 axi-cel trial, 8% (9/111) enrolled patients did not receive CART due to progressive disease or serious adverse reactions following leukapheresis (144). 14% (48/344) of the enrolled patients in the liso-cell trial had lymphoma complications or died before receiving CART, even with allowance for bridging therapy between leukapheresis and CART infusion (145). Similarly, 4% (3/74) of patients for whom KTE-X19 CART were successfully manufactured did not receive therapy (146), and ~8% (11/140) did not receive the manufactured ide-cel product (148). Combining the manufacturing failure and pre-infusion drop-off rates means ~9-30% of patients in CART clinical trials fail to receive the manufactured product (**Table 1**). Comprehensive data showing the real-world success of manufacturing commercial CART products in patients treated outside of a clinical trial is still pending, but the challenges of patient selection outside of the clinical trial setting, increasing adoption of commercial CART therapies at more cancer treatment centers, and additional challenges related to increased production and demand raises concerns whether or not these rates will increase.

TIL therapies face similar challenges in efficient manufacturing. Using the selected TIL manufacturing protocol with the traditional pre-REP, selection, and REP process typically takes 5-8 weeks for production, and the final expansion products are given immediately so as to avoid the risk of product loss due to cryopreservation (162). Combining the inability to grow TILs in ~20-25% of patients with the risk of disease progression during the long expansion times, drop-out rates in clinical trials initially ranged from 25-70% (156, 204–206). Use of a newer expansion method called the young TIL protocol has reduced manufacturing times by up to 3 weeks, but left the issue of concern with cryopreservation, limiting the ability to streamline the process with centralized expansion and direct distribution to clinical sites. Newer Gen-2 and Gen-3 versions of the commercially produced lifileucel LN-144/LN-145 TIL therapy seek to address these limitations. The Gen-1 TIL manufacturing time, which included phenotype selection, was 38 days in duration, and yielded a fresh, hypothermic product. The Gen-2 TIL process omits TIL selection and allows for production of a final, shippable, cryopreserved product within 22 days. Newer Gen-3 versions of LN-145 now seek to reduce the manufacturing time to just 16 days (207).

#### Improving ACT Therapy With PI3K Inhibitors

Taking into consideration the signaling cascade linking the CAR and TCR with PI3K signaling, the role of PI3K on T-cell differentiation and metabolic reprogramming, and the standard methods of expanding CARTs and TILs *ex vivo* using anti-CD3/CD28 stimulation, the evidence for utilizing PI3K-δ and -γ inhibition as a means to improve the ACT manufacturing process is compelling.

**Figure 5:**
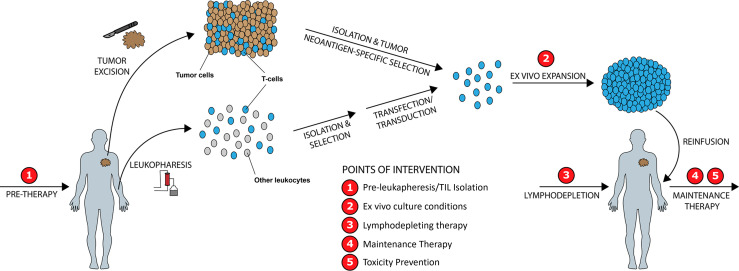
Improving ACT Therapy with PI3K-δ and PI3K-γ Inhibition. Five distinct timepoints during ACT manufacturing are identified wherein intervention with PI3K-δ/-γ inhibitors may improve manufactured product quality and clinical outcomes.

We identify five points of intervention within the ACT therapy manufacturing process wherein strategic changes can be made to modify intrinsic and extrinsic T-cell product characteristics and improve clinical and surrogate outcomes, in particular those related to prior therapy and cancer mediated T-cell differentiation, CD4/CD8 expansion ratios, Treg activity, T-cell exhaustion, and post treatment toxicity (**Figure 5**). These points of intervention include: **1)** *In vivo* treatment with a PI3K inhibitor (PI3Ki) therapy prior to leukapheresis or T-cell isolation, **2)** *Ex vivo* culture conditions, **3)** Lymphodepleting therapy, **4)** Maintenance therapy, and **5)** Toxicity prevention strategies. We discuss the exciting current preclinical and clinical data supporting the role of PI3K-δ and -γ inhibition in each of these situations while giving consideration to other non-PI3K mediated strategies currently being studied.

##### In Vivo PI3Ki Therapy Prior to Leukapheresis or T-Cell Isolation

The potential benefits to *in vivo* PI3K therapy prior to leukapheresis or T-cell isolation may have direct impacts on having a more favorable baseline T-cell phenotype for CART or increased T-cell yield from tumor for TIL therapy.

##### Altering the Pre-Leukapheresis T-Cell Phenotype

As previously discussed, the effects of prior systemic therapy and malignancy-driven T-cell terminal differentiation together reduce frequencies of memory and naïve cells in the leukaphereses T-cell product, thus reducing expansion capability and subsequent manufactured product response rates. As previously discussed, data from Fraietta et al. in CLL patients associated increased frequencies of CD27+CD45RO-CD8+ memory-like T-cells prior to CART manufacturing with improved disease remission rates (68). This finding supports exploring strategies to enhance memory-like T-cell expansion *in vivo* prior to pheresis with PI3Ki therapy. Such studies are planned but have yet to be undertaken. The majority of evidence to test this hypothesis derives from *ex vivo* pre-clinical and clinical studies and will be further discussed in the following sections.

##### Enhanced Intratumoral Infiltration

In TIL therapy, early studies noted high patient dropout rates of ~33% due to lack of T-cells in the tumor for successful expansion. These studies employed 2-phase TIL expansion protocols comprised of an initial pre-rapid expansion phase wherein tumor tissues are cultured with IL-2 to support T-cell division and survival, and a subsequent process of T-cell selection based on tumor reactivity followed by 14-days of rapid expansion (REP). During REP, T-cells were activated *via* anti-CD3 binding and co-cultured with irradiated feeder cells (either autologous or allogeneic) (208, 209). Challenges with this process include poor expansion during the pre-REP phase if no T-cells are present, lack of tumor-reactive T-cells to undergo rapid expansion, and reduction in T-cell health associated with rapid expansion and prolonged *ex vivo* culture (210). Therefore, methods to augment T-cell infiltration into the tumor microenvironment prior to T-cell isolation could therefore have positive effects on improving TIL manufacturing success. As prior noted, *in vivo* preclinical studies have demonstrated that PI3K-δ inhibition and knockout favorably increase the CD8+ TIL/Treg ratios in mouse models of lung, breast, and colon cancer (36). Not surprisingly, clinical studies are now underway to further assess such findings and characterize a role for PI3K inhibition prior to TIL isolation (NCT04142554 & NCT02646748).

##### Ex Vivo Culture Conditions

During *ex vivo* culture, preferential expansion of either the CD4+ cells vs CD8+ cells can skew CD4/CD8 ratios in infused product and influences the CART product persistence and efficacy (211). Concomitant stimulation of CD8+ TCRs and CARs promotes CD8+ CART-cell exhaustion with increased PD-1 and LAG-3 surface expression and decreased long-term persistence (212). Such divergences in maturation may also persistent following infusion, as T-cell phenotypes from the bone marrow of multiple myeloma patients post anti-BMCA-CART demonstrated increased frequencies of CD8+ Tscm and Tcm populations vs CD4+ Tcm and Te populations, suggesting CD4 and CD8 cells may undergo divergent courses of maturation *in vivo* following infusion (213). One approach, explored to counter this effect is to engineer paired CD4/CD8 CART products and infuse a final 1:1 therapy. Use of this strategy in a non-commercial anti-CD-19 CART product in a phase 1 trial of children and young adults with relapsed or refractory B-ALL reported a ~90% remission rate with 93% manufacturing success (NCT02028455) (214). These results are not uniform, however, as 25 patients in the Transcend NHL 00 Liso-cel trial received non-conforming CART products that did not contain equivalent CD4+/CD8+ product ratios (145). An alternate thought is that the presence of CD4+ cells beneficially influence and support CD8+ cell expansion. *Ex vivo* studies using pheresis samples from healthy donors and lymphoma patients demonstrated that initial CD4:CD8 ratios of at least 40%:60% more than doubled CD8+ CART expansion yields. A final 1:1 product was most closely achieved with starting ratios of 70%:30%, and this product also demonstrated the greatest anti-tumor effect against Raji (human B-lymphocyte cell line) lymphoma cells in an immunodeficient mouse model (215).

Growing separate CD4 and CD8 cultures or manipulating pre-expansion CD4/CD8 ratios introduces additional technical challenges. As an alternative, pharmacological manipulation of culture conditions may offer a simpler approach. Several groups, including our own, however, have shown that adding PI3K inhibitors during CART manufacturing may be an alternative to separately manufacturing CD4+ and CD8+ CART products. The Paulos lab has shown that PI3K-δ activity alters T-cell differentiation in murine and human CD8+ adoptively transferred T-cells using both murine transgenic TCR pmel-1 CD8+ T-cell models and a human peripheral blood T-cell model wherein T-cells are transduced with a tumor antigen specific CAR that recognizes the mesothelin, 4-1BB, and CD3ζ signaling domains (mesoCAR) (216). Inhibition of PI3K-δ during *ex vivo* manufacturing for 7-days with idelalisib resulted in a less differentiated T-cell products possessing a Tcm phenotype (increased CD62L/CCR7, CD127, and Tcf7) (216). These T-cell products also demonstrated increased anti-tumor activity against melanoma and mesothelioma in mice.

Phenotypically, altering PI3K signaling during *ex vivo* expansion appears to reduce T-cell exhaustion. We and others have shown PI3K inhibitors promote dose-responsive decreases in the expression of immune checkpoint molecules and exhaustion markers like TIM-3, LAG-3, and PD-1, thus restoring the Tcm phenotype (42, 113, 217). In the mesoCAR-T model, treatment with eganelisib (PI3K-γ) reduced surface TIM-3 expression (113). Altering the exhaustion phenotype of the manufactured CART product may have significant clinical implications, since in JULIET 11 patients with the highest percentages of LAG-3+ T-cells did not respond to tisagenlecleucel or had early relapse (142). Similarly, TILs from patients with HNSCC (head and neck squamous cell cancers) refractory to anti-PD-1 therapy demonstrated an enhanced exhaustion phenotype with TIM-3 upregulation that appears to be mediated by PI3K-Akt pathway activity (218). Further supporting these findings, the Paulos lab demonstrated that TIL cultures expanded from patients with lung carcinoma demonstrated reduced TIM-3 expression and higher CD62L when the cells were subsequently cultured with idelalisib for 2 weeks (216).

Functionally, PI3Ki-expanded CART cells demonstrate increased cytotoxicity, superior persistence and *in vivo* expansion, and greater anti-leukemia activity against human CLL cells engrafted in an immunodeficient NOG (NOD/Shi-scid/IL-2Rγ^null^) mouse model (42). In these experiments, short-term exposure to PI3Ki during CART cell manufacturing led to persistent alterations in CART cell function, even weeks after *in vitro* exposure to duvelisib, suggesting critical transcriptional and epigenetic changes occur in PI3Ki-expanded T-cells. Such changes may have lasting implications on cytotoxic function. *In vivo* mouse model studies with idelalisib demonstrated increased mesoCART-cell persistence up to 55 days post T-cell transfer, with the percentage of CD45+ peripheral blood lymphocytes almost 3-fold higher in the idelalisib cultured group vs control cultures (216). Pmel-1 transgenic CD8+ T-cells expanded *ex vivo* with either idelalisib (PI3K-δ) or eganelisib (PI3K-γ) showed enhanced *in vivo* tumor control against a B16F10 melanoma (median survival 70 days and ~60 days, respectively) vs control CD8+ cultures (30 days) (113).

Mechanistically, as previously reviewed, TCR stimulation with anti-CD3/CD28 beads during CART-cell manufacturing can induce PI3K/AKT signaling and T-cell terminal differentiation (68), similar to how PI3K/mTOR signaling is differentially regulated during antigen-driven expansion of CD4+ and CD8+ T-cells. Western blot analysis of murine CD8+ T-cells transduced with a TCR against the cancer-testis PLAC1 prior to in vivo infusion demonstrated that the transduced cells expressed increased PI3K-γ and phospo-AKT (219). Furthermore, lymphocyte differentiation studies have shown that daughter T-cells with increased PI3K/mTOR signaling differentiate into an effector cells, while those with reduced PI3K/mTOR signaling retain self-renewal capacity (220, 221). We also believe that minimal negative effects seen on ex vivo T-cell expansion in the presence of PI3K inhibition is mediated by concomitant signaling through the MEK/ERK pathway. We have previously shown that CART products cultured and expanded in the presence of duvelisib simultaneously demonstrated reduced phosphorylation of downstream PI3K pathway proteins with increased MEK and ERK phosphorylation (42).

A plausible explanation for the increased cytotoxicity seen in PI3Ki-expanded CART cells is secondary to downstream increases in CD27 and CD28 expression, a feature of T-cells noted to have increased *in vivo* persistence and cytotoxic activity (222). It has been well characterized that rapid expansion of TILs with IL-2 reduces T-cell surface CD27 and CD28 expression and results in an increased fraction of terminally differentiated T-cells in the final product (223, 224). In more recent years, cells expanded *via* the young TIL method has mitigated the adverse effects of rapid expansion, with young TIL products demonstrating longer telomeres and higher expression of CD27 and CD28 (210, 225). Pharmacologically achieving similar results with PI3Ki, however, may be less technically challenging. Already Dwyer et al. has shown that mesoCART cultured with eganelisib demonstrate reduced T-cell differentiation and increased CD27/CD28+ surface expression (113). Inhibition of PI3K-δ, either alone or simultaneously with PI3K-γ, also increased CD28 expression in these models.

Taken together, PI3K inhibition during *ex vivo* T-cell culture has the ability to inhibit differentiation and exhaustion mechanisms without affecting proliferation. Further supporting these preclinical and clinical correlate studies is data from a recent phase I study of bb21217, an anti-BCMA CART therapy based on ide-cel that adds the PI3K inhibitor bb007 during *ex vivo* culture to enrich the drug product for memory-like T-cells (226). Comparison of T-cell populations from peripheral blood versus the manufactured drug product showed bb21217 had increased enrichment for CD27+/CCR7+ Tm cells, depletion of CD57+ senescent cells, increased CD127 expression (a marker of persistent Tm formation), and higher peak *in vivo* CART expansion.

As prior discussed, prolonged manufacturing time and manufacturing failure remain as major clinical hurdles. While data is lacking, early studies promising suggest the addition of PI3Ki to ACT expansion cultures can shorten manufacturing times or reduce the manufacturing failure rate.

##### Lymphodepleting Therapy

As previously discussed, lymphodepletion with chemotherapy prior to CART infusion improves T-cell persistence and cytotoxic activity (186) by killing immunosuppressive Tregs and MDSCs, eliminating homeostatic cytokine sinks (187), and modulating tumor IDO (indoleamine 2,3-dioxygenase) (188). Interestingly, PI3K-δ inhibition with idelalisib in patients with CLL has been shown to inhibit CD4+CD25+CD127- Treg proliferation and Treg-induced suppression of CD4+ and CD8+ T-cells, suggesting pre-infusion pharmacologic PI3Ki therapy may favorably augment the effects of lymphodepletion (121). Multiple studies in TIL therapy have already been performed to add additional therapy, like radiation, to the to the pre-conditioning regimen (227–229), but none with PI3K inhibitors.

##### Maintenance Therapy

Another consideration is to continue PI3Ki therapy following ACT product infusion. One reason for this is to mitigate the ill effects of rapid *in vivo* expansion. Similar to *ex vivo* anti-CD3/CD28 stimulation, rapid *in vivo* CART expansion induces increased expression of the T-cell exhaustion marker PD-1. Adding anti-PD-1/PD-L1 therapy after ACT is one approach being considered to rescue T-cells from exhaustion, but responses from CART trials have not been uniformly positive (230). Multiple clinical trials are now studying post-TIL anti-CTLA-4 or anti-PD-1 therapies in advanced or metastatic cutaneous melanoma, HNSCC, NSCLC, or cervical cancers (NCT02278887, NCT03645928, NCT03108495). Post-TIL HD-IL-2 therapy, which is given to support TIL expansion and persistence, may also unfavorably stimulate *in vivo* Treg expansion. In one study, melanoma patients with the highest fold expansions of these ICOS+ Treg-like cells following TIL therapy and HD-IL-2 were noted to have worse clinical outcomes than patients with fewer ICOS+ Tregs (231). Taken together, post ACT PI3Ki therapy, with or without checkpoint blockade, may favorably modulate cytotoxic T-cell and suppress Treg expansion post ACT.

#### Toxicity Prevention

Reducing toxicities like CRS would also improve outcomes with CART. While tocilizumab and steroids are useful treatments for CRS, tocilizumab requires IV infusion and steroids can inhibit the CART effect. There remains much need for safe and tolerable therapies to use in the post-infusion period to reduce risk of CRS without affecting CART efficacy. Recent preclinical evidence suggests that in *ex vivo* CRS assays and *in vivo* murine models of CD19 CART, the dual PI3K-δ and -γ inhibitor duvelisib, antagonizes and reduces IL-6 secretion better than single agent isoform selective PI3K-δ and -γ inhibitors without negatively inhibiting CART function (232). More data from pre-clinical humanized mouse models and from the clinical setting are necessary to further validate and test these promising approaches.

## Clinical Translation And Future Directions

Despite the recent success of T-cell immunotherapies, much room for improvement remains. With ACT, the timing of cell manufacturing, availability of normal lymphocytes and tumor antigen for ACT manufacturing, and the complexity of the manufacturing process must be intimately integrated with the clinical treatment timeline to achieve success. This poses unique challenges, and we have highlighted herein five points in the ACT manufacturing process where the addition of PI3K inhibitors might result in improvement in manufactured product or the clinical outcome of ACT. T-cell targeted immunotherapies face challenges related to immune cell infiltration and T-cell activity in the TME. Multiple therapeutic synergies pairing anti-PD/PD-L1 agents with chemotherapy, radiation therapy, molecular targeted therapies like HER-2 targeted agents and inhibitors of VEGF, CDK4/6, PARP, HDAC, BRAF, MEK, and other checkpoint inhibitors like TIM-3 and LAG-3 (NCT04370704) have been considered in order to overcome the well-characterized challenges of tumor immune escape mechanisms (95, 233–236). Furthermore, targeting metabolic pathways of cancer and related cells in the TME contributing to nutrient and metabolic stress has become an area of growing interest with multiple therapeutic targets under consideration, including IDO, MCT1/MCT4, mitochondrial complex I, and the mitochondrial tricarboxylic acid (TCA) cycle (237–240).

### Toxicities With PI3K Inhibitors

While the PI3K pathway is critical to cancer development and regulates activity of multiple immune cell populations in the TME, clinical development of pan-PI3K inhibitors for the treatment of solid tumor malignancies in melanoma, breast, lung, colorectal, and head and neck cancers has faced challenges of inefficacy and clinical toxicity (16–23).

The toxicities of PI3K inhibitors must be considered during clinical applications. Clinically, they can manifest with autoimmune or autoinflammatory signatures, including liver toxicity, hyperglycemia, rash, and colitis. Notably, these symptoms overlap during treatment of both solid and hematologic malignancies, vary by route of administration, and appear to improve with isoform-selectivity (241, 242). Furthermore, while the mechanisms of immunomodulation governing these toxicities are still yet to be fully characterized, many of them toxicities overlap with those seen with ICIs, which must be further considered during synergy trials (78).

Isoform selectivity and drug formulation have a direct effect on clinical side-effects and toxicities (241, 242). Hyperglycemia is most commonly seen with PI3K-α inhibition (selective and pan-inhibitors), due to therapy induced disruptions in insulin signaling and glucose hemostasis leading to clinically evident metabolic changes (243). Comparison of idelalisib (oral, PI3K-δ) with copanlisib (IV, PI3K-α/δ) shows that Grade 3 diarrhea or colitis is rarely seen with copanlisib, but common (>15%) with idelalisib, likely due to the IV formulation (241). Furthermore, severe colitis and pneumonitis risk is higher with PI3K-δ and -γ inhibition (idelalisib, copanlisib, and duvelisib) (31, 242, 244).

Clinical trials have shown PI3K-δ and -γ inhibition are associated with increased immune suppression and opportunistic infection risk (111, 244). In DYNAMO, a Phase II study of duvelisib in patients with refractory indolent non-Hodgkin lymphoma, dual -δ/-γ inhibition led to frequent AEs of any-grade of diarrhea (48.8%), nausea (29.5%), neutropenia (28.7%), fatigue (27.9%), and cough (27.1%) (244). The most frequent grade 3/4 AEs were neutropenia (24.8%), diarrhea (14.7%), anemia (14.7%), and thrombocytopenia (11.6%) and similar rates of grade 3 neutropenia (27%) were observed in a Phase I trial of the PI3K-δ selective idelalisib, also in patients with relapsed non-Hodgkin lymphoma (29).

The combination of a greater understanding of the redundant and non-redundant functions of PI3K signaling in T-cells, the systemic toxicities of targeting different isoforms, and the broad availability of isoform selective agents, has initiated a new wave of clinical testing, particularly with using PI3K-α inhibitors for *PIK3CA* mutated cancers (245, 246), PI3K-β in cancers with PTEN loss (247, 248), and PI3K-δ and -γ inhibitors as cancer immunotherapies and T-cell immunomodulators.

### Ongoing Clinical Investigations

Clinical evaluation of PI3K-δ and -γ inhibitors as immunomodulators of T-cell activity, metabolism, and the TME is still in the early stages, but multiple efforts are underway. A search of actively recruiting or pending trials in ClinicalTrials.Gov related to “PI3K” and “Cancer” was conducted. 177 studies were identified that were either recruiting, not yet recruiting, or active/not recruiting. Attention was given to trials incorporating inhibitors of PI3K-δ and -γ with T-cell targeted immunotherapies. We excluded trials for cancer specific mutations in *PIK3CA*, *PIK3CB*, *AKT*, or *PTEN* and trials for inhibitors of PI3K-α and -β (alpelisib, taselisib, serabelisib, HS-10352, GSK2636771), AKT (ipatasertib, AZD5363), mTOR (everolimus, TAK-228), or dual PI3K/mTOR (gedatolisib, samotolisib, HEC68498, paxalisib).

We identified 18 clinical trials combining copanlisib, duvelisib, eganelisib, idelalisib, parsaclisib, SF1126, and TGR-1202 with checkpoint inhibitors of PD-1 or PD-L1 in solid and hematologic malignancies (**Table 3**). Particularly interesting are trials incorporating expression of biomarker to clinically assess immune cell changes following PI3K-δ and -γ inhibition. Of note, 7 trials utilize PI3K-α or -α/-β targeting inhibitors. Of these trials, 4 are in solid tumor indications, and in the absence testing for mutations, amplifications or gene loss in PI3K or PTEN, the primary motive for these is to investigate the -δ and -γ effects.

**Table 3:** Current clinical trials combining PI3K-δ and -γ inhibitors with T-cell targeted therapies.

Indication	PI3K Inhibitor	Synergy Drug	PI3K Isoform	Trial Phase	NCT Number	Immune Cell Biomarker	Solid or Hematologic
HNSCC, NSCLC, CRC, HCC	Copanlisib	Nivolumab	alpha and delta	Phase 1	NCT03735628	No	Solid
Unresectable or Metastatic MSS Solid Tumors	Copanlisib	Nivolumab	alpha and delta	Phase 1/2	NCT03711058	No	Solid
Indolent Lymphoma	Copanlisib	Nivolumab + Rituximab	alpha and delta	Phase 1	NCT04431635	No	Hematologic
R/R DLBCL and R/R PMBCL	Copanlisib	Nivolumab	alpha and delta	Phase 2	NCT03484819	Yes	Hematologic
Richter’s Transformation or Transformed Indolent Non-Hodgkin’s Lymphoma	Copanlisib	Nivolumab	alpha and delta	Phase 1	NCT03884998	Yes	Hematologic
Ann Arbor Stage III/IV Lymphoma, Metastatic/Recurrent Malignant Solid Neoplasm	Copanlisib	Ipilimumab + Nivolumab	alpha and delta	Phase 1	NCT03502733	No	Both
PD-1 refractory unresectable melanoma	Duvelisib	Nivolumab	delta and gamma	Phase I/2	NCT04688658	Yes	Solid
Stage IIB-IVB Mycosis Fungoides and Sezary Syndrome	Duvelisib	Nivolumab	delta and gamma	Phase 1	NCT04652960	Yes	Hematologic
Richter Syndrome or Transformed Follicular Lymphoma	Duvelisib	Nivolumab	delta and gamma	Phase 1	NCT03892044	Yes	Hematologic
Advanced Solid Tumors, NSCLC, Melanoma, HNSCC, TNBC, Adrenocortical Carcinoma, Mesothelioma, High-circulating MDSCs	Eganelisib (IPI-549)	Nivolumab	gamma	Phase 1	NCT02637531	Yes	Solid
ICI naïve, platinum refractory UCC	Eganelisib (IPI-549)	Nivolumab	gamma	Phase 2	NCT03980041	No	Solid
Breast Cancer, RCC	Eganelisib (IPI-549)	Atezolizumab	gamma	Phase 2	NCT03961698	Yes	Solid
NSCLC	Idelalisib	Pembrolizumab	delta	Phase 1/2	NCT03257722	Yes	Solid
R/R CLL, R/R low-grade B-cell NHLs	Idelalisib	Pembrolizumab	delta	Phase 2	NCT02332980	Yes	Hematologic
CRC, Endometrial Cancer, Melanoma, Head and Neck Cancer, Lung Cancer, MMR-deficient Tumors, Breast Cancer, Pancreatic Cancer, RCC, Solid Tumors, UC	Parsaclisib	Pembrolizumab	delta	Phase 1	NCT02646748 (Group B)	Yes	Solid
Unresectable or Metastatic Solid Tumors	Parsaclisib	Retifanlimab (anti-PD-1)	delta	Phase 1	NCT03589651	No	Solid
Advanced HCC	SF1126	Nivolumab	alpha, beta, delta, and gamma*	Phase 1	NCT03059147	No	Solid
CLL, B-cell NHL	TGR-1202	Pembrolizumab	delta	Phase 1	NCT03283137	No	Hematologic

NSCLC (Non-small Cell Lung Cancer); HNSCC (Head and Neck Squamous Cell Carcinoma; TNBC (Triple negative breast cancer); CLL (Chronic Lymphocytic Leukemia); CRC (Colorectal Cancer); HCC (Hepatocellular Carcinoma), RCC (Renal Cell Carcinoma); UC (Urothelial Carcinoma), NHL (Non-Hodgkin’s Lymphoma), MSS (Microsatellite Stable), R/R (relapsed/refractory); DLBCL (diffuse large B-cell lymphoma); PMBCL (primary mediastinal large B-cell lymphoma).

*Bromodomain-4 and PI3K inhibitor.

NCT04688658 is a phase I/II trial in melanoma studying changes in immune cell function from tumor and peripheral blood prior to and while on duvelisib therapy. MARIO-3 (Macrophage Reprogramming in Immuno-Oncology) (NCT03961698) incorporates pre- and on-treatment tissue biopsies to correlate the effects of eganelisib on PD-L1 expression in tumor-infiltrating immune cells. In hematologic malignancies, NCT03484819 will characterize the effects of the copanlisib and nivolumab combination regimen on tumor cells, tumor microenvironment and the immune response in relapsed/refractory DLBCL and Primary Mediastinal Large B-cell Lymphoma. NCT03502733 will report changes in PD-1, PD-L1, PD-L2, and immune cell profiles and markers of immune-modulation in CLL and low-grade B cell NHL patients with copanlisib and combination ICI.

Two trials (NCT02646748 and NCT03257722) are combining the PI3K-δ inhibitors parsaclisib and idelalisib, respectively, with pembrolizumab to assess the effects on Tregs. In NCT02646748, pre- and post- therapy changes in the total number of tumor infiltrating lymphocytes and ratio of CD8+/FoxP3+ Tregs will be measured. NCT03257722 will use Treg suppression (target of 80% suppression in 80% of patients) to calculate the optimal idelalisib immunomodulatory dose. The latter trial design raises an important consideration of incorporating meaningful biomarker driven correlates into clinical trials to facilitate identification of the optimal immunomodulatory dose versus maximally tolerated dose.

Macrophage remodeling with PI3K-γ inhibitors is also of interest. NCT03795610 is a window of opportunity study in HNSCC to determine whether 3 weeks of the PI3K-γ inhibitor eganelisib prior to surgery is sufficient to drive macrophage phenotype switching in tumors. NCT02637531 is a multi-group study combining eganelisib with nivolumab, in which one group is stratified by the presence of high-circulating peripheral blood MDSCs. Similarly, Mario-275 (NCT03980041) evaluates eganelisib with nivolumab in patients with advanced urothelial carcinoma and incorporates MDSC levels as a part of its randomization process.

Taken together, our current knowledge combined with the results of these trials and future studies has the potential to drastically change how we re-purpose PI3K inhibitors, in particular PI3K-δ and -γ inhibitors, as immunomodulatory agents. While current focus heavily remains on T-cell activation, differentiation, and anti-tumor activity persistence and memory in developing immunotherapies, we anticipate future investigations focusing on immune cell metabolism and other immune cell suppressive functions in the TME will yield biologically meaningful and clinically significant changes in the field of immuno-oncology.

## Author Contributions

SC conceived, drafted, wrote, and revised the manuscript and accompanying figures and tables. CF assisted with writing and developing figures. TK developed and revised the figures and tables. CP critically reviewed and revised the manuscript regarding TIL therapy and immune checkpoint blockade. MS critically reviewed and revised the manuscript regarding T-cell metabolism and differentiation. EW was involved with drafting and critically reviewing and revising the manuscript. All authors contributed to the article and approved the submitted version.

## Funding

SC: NIH T32CA160040, ECOG-ACRIN Paul Carbone, MD Fellowship Award; EW: NIH 5R01AI145231-03; MS: NIH 1R01CA208328-01A1, LLS (Leukemia/Lymphoma Society) Award # 6573-19.

## Conflict of Interest

The authors declare that the research was conducted in the absence of any commercial or financial relationships that could be construed as a potential conflict of interest.

## Publisher’s Note

All claims expressed in this article are solely those of the authors and do not necessarily represent those of their affiliated organizations, or those of the publisher, the editors and the reviewers. Any product that may be evaluated in this article, or claim that may be made by its manufacturer, is not guaranteed or endorsed by the publisher.
